# 370. Swimming Against the Current: Outcomes of Standard versus Modified Outpatient Parenteral Antimicrobial Therapy (OPAT) Services

**DOI:** 10.1093/ofid/ofae631.111

**Published:** 2025-01-29

**Authors:** Evelyn Villacorta Cari, Armaghan-E Rehman Mansoor, Alice C Thornton, Alisha Clemons, Ryan P Mynatt, Ashley Logan, Donna R Burgess, Jaime Soria

**Affiliations:** University of Kentucky, Lexington, KY; University of Kentucky, Lexington, KY; The University of Kentucky, Lexington, Kentucky; University of Kentucky, Lexington, KY; University of Kentucky, Lexington, KY; University of Kentucky HealthCare, Lexington, Kentucky; UK HealthCare, Lexington, KY; University of Kentucky, Lexington, KY; University of Kentucky, Lexington, KY

## Abstract

**Background:**

OPAT programs enable intravenous (IV) antibiotic treatment outside the hospital, either at home or in an outpatient facility. We established institutional OPAT criteria to identify patients who could safely complete OPAT at home (standard, or S-OPAT) and those with potential barriers (modified, or M-OPAT). The study aims to compare outcomes in both groups.

**Methods:**

We conducted a retrospective cohort study analyzing data collected via OPAT program operations from July 2019 to March 2024. These data were collected using the REDCap electronic data capture system. Statistical analyses were performed with Stata 17.

**Results:**

The analysis included 3,344 patients referred to the OPAT program, with 2,746 (82.1%) assigned to the S-OPAT group and 598 (17.9%) to the M-OPAT group. The median age was similar in both cohorts, and Male patients were 1,583 (58.9%) in S-OPAT and 389 (65.1%) in M-OPAT (p = 0.001). The most prevalent infection treated in both groups was Osteomyelitis: 535 (19.5%) patients in S-OPAT and 156 (26.1%) patients in M-OPAT (p< 0.001), followed by bacteremia at 484 (17.6%) patients in S-OPAT and 107 (17.9%) in M-OPAT (p= 0.859).

A history of IV drug use was reported in 108 (4.0%) patients of S-OPAT and 200 (33.4%) patients of M-OPAT. M-OPAT patients reported barriers such as lack of social support (homelessness, inability to administer antibiotics at home, or requiring additional medical care) in 422(70.6%) patients, incarceration in 58 (9.7%) patients, and antibiotics received during dialysis sessions in 58 (9.7%) patients.

Reported outcomes, including clinical improvement, were not significantly different between these groups [S-OPAT: 2367 (86.2%) vs. M-OPAT: 530 (88.8%); (p=0.11)]. Treatment completed as expected, extended, or discontinued were as follows: S-OPAT 2106 (76.7%) vs. M-OPAT 486 (81.3%), (p=0.015); S-OPAT 398 (14.5%) vs. M-OPAT 67 (11.2%), p=0.037; and S-OPAT 237 (8.6%) vs. M-OPAT 43 (7.2%), p=0.289.

**Conclusion:**

Despite the higher prevalence of social and medical challenges in the M-OPAT cohort, treatment outcomes, including clinical improvement and completion rates, were comparable to those in the S-OPAT group; the study highlights the feasibility and effectiveness of outpatient antibiotic treatment, even in patients facing significant barriers to care.

**Disclosures:**

**Alisha Clemons, APRN**, Gilead Sciences: Honoraria

